# Liposome Photosensitizer Formulations for Effective Cancer Photodynamic Therapy

**DOI:** 10.3390/pharmaceutics13091345

**Published:** 2021-08-27

**Authors:** Sherif Ashraf Fahmy, Hassan Mohamed El-Said Azzazy, Jens Schaefer

**Affiliations:** 1 Department of Chemistry, School of Sciences & Engineering, The American University in Cairo, AUC Avenue, P.O. Box 74, New Cairo 11835, Egypt; sheriffahmy@aucegypt.edu; 2 School of Life and Medical Sciences, University of Hertfordshire Hosted by Global Academic Foundation, R5 New Garden City, New Capital AL109AB, Cairo 11835, Egypt; 3 Department of Pharmaceutics and Biopharmaceutics, University of Marburg, Robert-Koch-Street 4, 35037 Marburg, Germany

**Keywords:** photodynamic therapy, photosensitizers, liposomes, stealth liposomes, thermosensitive liposomes, tetraether lipids, cancer

## Abstract

Photodynamic therapy (PDT) is a promising non-invasive strategy in the fight against that which circumvents the systemic toxic effects of chemotherapeutics. It relies on photosensitizers (PSs), which are photoactivated by light irradiation and interaction with molecular oxygen. This generates highly reactive oxygen species (such as ^1^O_2_, H_2_O_2_, O_2_, ·OH), which kill cancer cells by necrosis or apoptosis. Despite the promising effects of PDT in cancer treatment, it still suffers from several shortcomings, such as poor biodistribution of hydrophobic PSs, low cellular uptake, and low efficacy in treating bulky or deep tumors. Hence, various nanoplatforms have been developed to increase PDT treatment effectiveness and minimize off-target adverse effects. Liposomes showed great potential in accommodating different PSs, chemotherapeutic drugs, and other therapeutically active molecules. Here, we review the state-of-the-art in encapsulating PSs alone or combined with other chemotherapeutic drugs into liposomes for effective tumor PDT.

## 1. Introduction

One of the fundamental challenges in designing successful tumor-targeting approaches is the selective delivery of anticancer drugs to cancerous cells. Although both the cancerous and healthy tissues are impacted by the cytotoxic effects of anticancer drugs, most targeting approaches depend on the fact that the rapidly proliferating cancer cells would be more affected by chemotherapeutics than healthy ones [1,2,3,4]. This warrants the development of novel targeted delivery systems capable of selectively eradicating cancerous cells. Various drug delivery vehicles and nanocarriers have been developed in this context, including polymeric and metal nanoparticles, supramolecular nanocapsules, liposomes, host-guest complexes, and nanofibers [1,2,5,6]. Multimodal systems were designed by exploiting the capability of the nanosystems to co-deliver chemotherapeutic drugs and targeting entities, resulting in more efficient treatment.

Photodynamic therapy (PDT) employs pharmacologically inactive photosensitizers activated upon exposure to light in the presence of oxygen. It is a non-invasive therapeutic approach that does not require sophisticated equipment or setup and has been employed to treat cancer, cardiovascular, and skin diseases [5,6,7,8,9,10,11,12,13,14,15]. This review presents the basic principles and current challenges of using PDT in cancer therapy and state-of-the-art approaches in formulating liposome photosensitizers to improve the therapeutic significance of PDT.

## 2. Photodynamic Therapy in Treating Cancer

**Figure 1:**
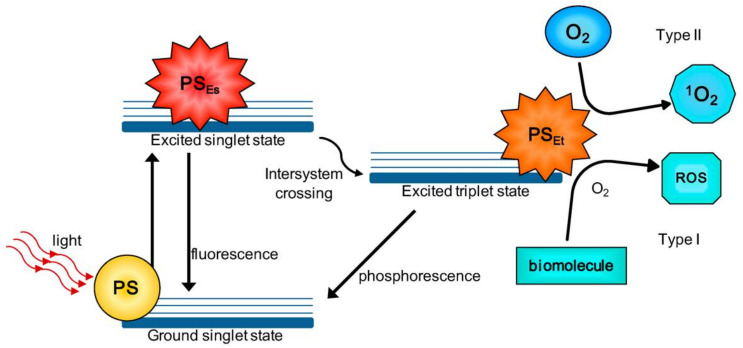
Mechanism of action of PDT demonstrating Type I and Type II reactions. PS_Es_, PS excited singlet state; PS_Et_, PS excited triplet state; ROS, reactive oxygen species. Reprinted with permission from ref. [26]. 2016 Calixto et al.

PDT was approved in several countries for treating cancer after its approval for recurrent bladder cancer treatment by the Canadian Health Protection Branch [1]. PDT depends on the interaction of a photosensitizer (PS), delivered to target tissues, and with the light of a specific wavelength in the presence of molecular oxygen dissolved in the cytoplasm [16,17,18,19,20,21,22]. Upon light absorption, the PS molecules are transferred from the ground state to an excited singlet state and then to a triplet excited state via the intersystem crossing. In the triplet excited state, the PS undergoes two simultaneous reactions (1) Type I electron transfer reactions which involve the direct reaction of PS with cell components forming anionic or cationic radicals that react with molecular oxygen generating ROS, and (2) Type II energy transfer reactions which involve direct reaction of PS with molecular oxygen producing singlet oxygen (^1^O_2_) (Figure 1). The generated highly reactive ROS (such as ^1^O_2_, H_2_O_2_, O_2_, ·OH) exert their cytotoxic effects via irreversible oxidation of the cellular and subcellular organelles and induce apoptosis or necrosis, leading to cell death. ROS can also induce autophagy by several mechanisms, leading to cytoprotective and cell killing responses [23,24,25].

**Figure 2:**
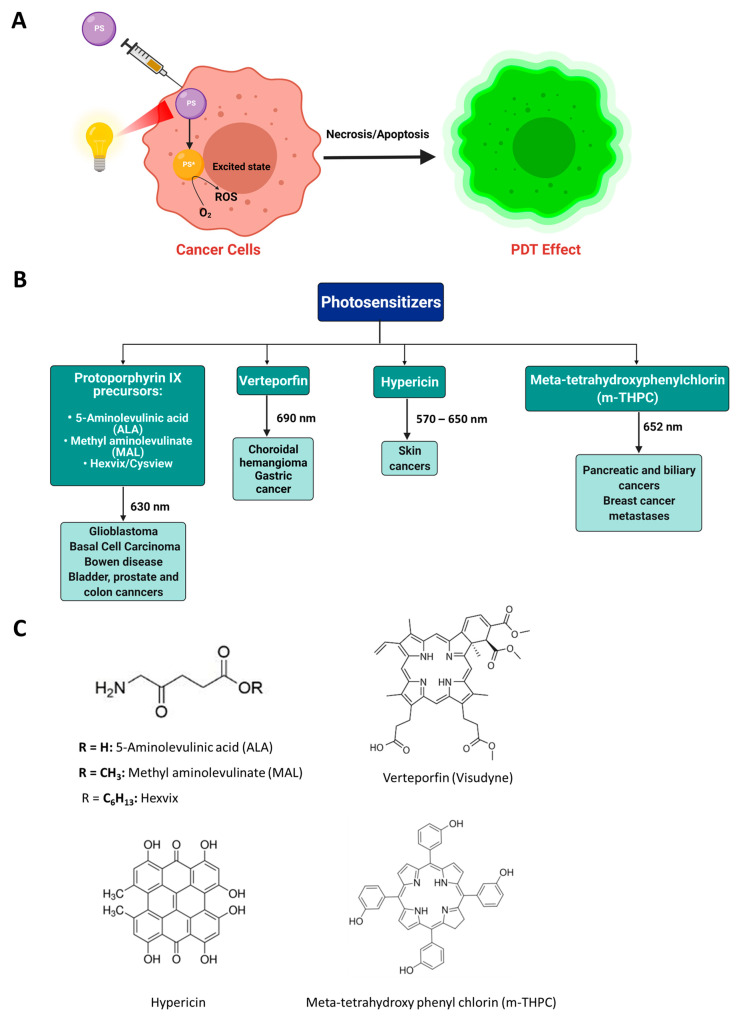
(**A**) General principle of photodynamic therapy; (**B**) FDA and/or EU-approved photosensitizers in cancer photodynamic therapy; (**C**) Chemical structures of approved photosensitizers. PS, photosensitizer; PS*, photosensitizer excited state.

Many parameters influence the effectiveness of PDT. These include the type and dose of PS, route of administration, intensity of light used, type of tumor, and concentration of dissolved cytoplasmic oxygen. The ideal PSs should be pharmacologically inactive in the absence of light irradiation, pure, water-soluble, and selectively present in tumor cells. It should have an absorption spectrum preferably between 650 and 800 nm and rapid elimination rates. Two classes of PSs exist: Porphyrin PSs (three generations) and non-porphyrin PSs [27]. First-generation PSs (such as hematoporphyrins) have been developed and undergone clinical trials for more than 40 years [16]. However, they suffered various drawbacks, including (i) poor tissue penetration, (ii) low chemical stability, (iii) activation when irradiated with wavelengths below 650 nm, (iv) causing skin hypersensitivity reactions, and (v) low elimination rates and long half-lives. Most of the issues attributed to hematoporphyrins refer mainly to Photofrin. This is a complex mixture of porphyrin dimers and higher oligomers, some of which persist in the skin and result in skin photosensitization. Second-generation PSs (such as metalloporphyrins, porphycenes, purpurins, chlorins, protoporphyrins) was developed to overcome most of these shortcomings [17]. 5-Aminolevulinic acid (ALA), methyl aminolevulinate (MAL; Metvix), and Hexvix/Cysview are precursors of protoporphyrin IX, which absorbs at 630 nm [28,29]. They were granted FDA approvals and are used to treat glioblastoma, basal cell carcinoma, Bowen disease, prostate, bladder, and colon cancers [30,31,32]. Meta-tetrahydroxy phenyl chlorin (m-THPC, temoporfin), excitation wavelength of ~652 nm, is approved in the EU to treat biliary and pancreatic cancers and breast cancer metastases. Verteporfin (Visudyne), a benzoporphyrin derivative (BPD), with an excitation wavelength of 690 nm, has been granted FDA approval to treat choroidal hemangioma and gastric cancer. Additionally, hypericin (excitation wavelength of 570–650 nm) is an FDA-approved PS for treating skin cancers (Figure 2) [33,34,35]. Recently, third-generation PSs have been developed by conjugation of second-generation PSs to biological targeting moieties, such as carbohydrates, peptides, or antibodies. This would enhance the selectivity of PSs and minimize undesired adverse effects [29,36]. Non-porphyrin PSs include psoralens, anthracyclines, chalcogenopyrylium dyes, cyanines, and phenothiazinium dyes. Despite the promising effects of PDT in cancer treatment, it still suffers from several shortcomings. These include poor biodistribution of hydrophobic PSs, poor cellular uptake, difficulty applying PDT to deep cancer tissues (hindering light penetration), and low selectivity to cancer cells [37,38]. In addition, cancer cells’ oxygenation is essential to achieving effective PDT. Because cancer tissues are bordered by necrotic cells, and compact tumor masses, it is challenging to treat deep cancers using the traditional PDT. Because of the low tissue penetration of visible light, PDT is only effective for treating superficial, and skin tumors [26]. Furthermore, using photofrin may cause long-lasting photosensitivity reactions. Consequently, encapsulation of PSs within different nanocarriers has been proposed to overcome traditional PDT’s shortcomings, such as improving water solubility, bioavailability, and selective targeting of PSs [39].

## 3. Liposomal Photosensitizer Formulations for Tumor Photodynamic Therapy

PSs have entirely or partially been encapsulated, conjugated, or immobilized onto different nanocarriers. Multimodal integration of nanocarriers, such as polymeric nanoparticles, silica nanoparticles, and nanofibers, with PSs, offers several advantages compared to traditional PDT [1,2,3,4]. These include: (i) increasing the solubility of hydrophobic PSs, and hence, improving their biodistribution, pharmacokinetics, and cellular uptake; (ii) maintaining constant release rates of PSs at the targeted tumor cells; (iii) the ability to selectively target high loading capacity enabled by the substantial surface-to-volume ratios of nanocarriers which facilitate their surface decoration with particular ligands that can target the overexpressed receptors and proteins in tumor tissues without off-target toxic effects; (iv) boosting the preferential accumulation of PSs into cancer cells via the enhanced permeability and retention (EPR) effect; and (v) expanding the clinical applications of PDT to include additional types of cancer [40,41,42,43,44,45,46,47,48].

**Figure 3:**
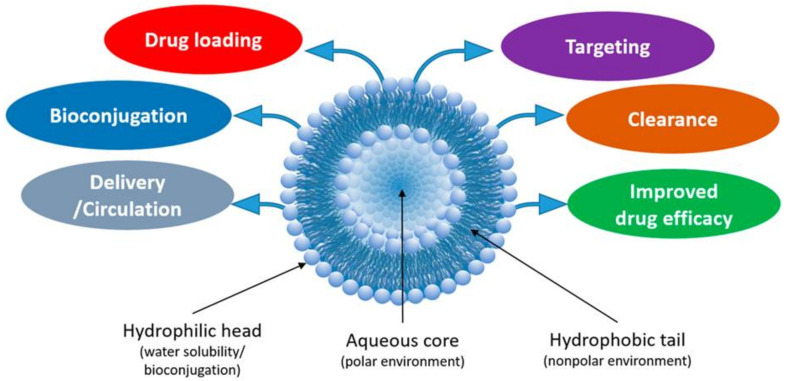
Liposomes are spherical vesicles with an aqueous inner core enclosed by one or more phospholipid bilayers which permit the conjugation of various functional groups. Reprinted with permission from ref. [49]. 2020 Almeida et al.

Liposomes are promising nanoplatforms that could be integrated with PDT to enhance the eradication of cancer cells without affecting healthy ones. They are spherical vesicles that consist of a hydrophilic head and a hydrophobic tail. They are self-assembled in an aqueous medium, with the assistance of hydrophobic interaction, developing a sealed structure formed of one or more lamella having the hydrophilic heads oriented towards the outer surfaces of the lamella and the hydrophobic tails founding the lamella interiors. This forms an aqueous core inside the liposomes (Figure 3) [49,50]. Liposomes are prepared using diverse types of naturally occurring phospholipids, which are biocompatible and biodegradable (Table 1). The lipid composition plays a vital role in the stability of liposomes in the systemic circulation, drugs encapsulation efficiencies, and drug release at tumor sites. For instance, the use of DSPC in the liposomal formulation results in improved encapsulation efficiency and stability compared to EPC and DPPC. This is because of the lengthy fatty acid chain of DSPC and the rigidity of the acyl chains of DSPC. Moreover, the use of cholesterol enhances liposomal stability and inhibits the undesirable drug in the systemic circulation. The inclusion of DPPE mPEG5000 in the lipid composition prolongs the blood circulation time of the liposomes, due to the additional steric hindrance created. This reduces liposomal uptake by the reticuloendothelial system. Moreover, the fusogenic features of some lipids (such as DPPG) in the liposomal membrane are reported to improve the ability of liposomes to cross the cancer cell membrane [51,52].

Liposomes have unique biological and physicochemical properties setting them apart from other nanoparticles. They are prepared by the self-assembly of phospholipids and/or tetraethers lipids which are biocompatible and biodegradable [44]. Moreover, liposomes can: (i) Accommodate hydrophilic and hydrophobic agents (such as proteins, nucleic acids, chemotherapeutics, and various PSs); (ii) be decorated and bioconjugated with various functional groups and surface targeting moieties (such as proteins, polymers, and peptides), which would improve their physicochemical properties, drug loading, clearance and/or trigger the release of their cargos into the target tissue; (iii) minimize the opsonization phenomenon by being less identifiable by the reticuloendothelial system leading to prolonging the half-life of the host molecules in the systemic circulation (>48 h) and facilitating the preferential passive accumulation inside tumor tissues; and (iv) widen the therapeutic index for most drugs (Figure 3) [44,45,46,47,48,49]. Liposomes can enter the cancer cells either by endocytosis or via membrane fusion. For instance, liposomes containing anionic phospholipids display faster endocytosis which augments their intracellular uptake. Moreover, liposomes containing fusogenic lipids demonstrate the ability to fuse and penetrate the cancer cell membrane. A recent publication examined the pathways of cellular internalization of liposomes [50].

It is worth mentioning that proteins in the circulation adsorb to liposomes administered systemically, leading to the formation of a protein corona that interacts with immunoglobulins, complement proteins, and phagocytes in the circulation. This would stimulate cytokine production in the tumor microenvironment leading to adaptive antitumor immunity. Moreover, the interaction of liposomes with serum proteins (specially opsonins) plays an essential role in the rapid clearance of liposomal by phagocytes in the blood, liver, and spleen. PEGylation slows the release of liposomes leading to prolongation of their half-lives [53,54].

Various liposomal formulations which improved the physicochemical properties and pharmacokinetics of cancer drugs have received FDA approval (Table 2). Similar liposomal formulations encapsulating PSs can be investigated for improved cancer PDT.

### 3.1. Liposomes for Photodynamic Tumor Therapy

Several liposomes encapsulating anticancer drugs and carrying targeting moieties (such as mab, glutathione, EndoTAG-1, and transferrin) are currently in clinical trials [55]. In PDT, liposomes can target the loaded PSs to cancer cells via active or passive mechanisms. Active targeting is achieved by decorating the liposomal surface with ligands (such as antibodies) that recognize and bind to overexpressed receptors and proteins in tumor tissues, such as folate, estrogen, spermine, and galactose receptors. Passive targeting takes place via the EPR effect. Blood vessels in healthy tissues are organized and tightly packed, which prevents the extravasation of liposomes. On the other hand, the blood vessels of tumor tissues are disorganized, due to the rapid proliferation of the vascular endothelium inside the tumor tissues [44,45,46,47,48,49,50,51,52,53,54,55,56]. Furthermore, the lymphatic drainage is impaired inside the tumor tissue, resulting in the overexpression of permeability mediators, such as bradykinin, nitric oxide, and prostaglandins, which increase EPR [56]. Consequently, nanoliposomes (100–300 nm) can passively cross the loose tumor endothelial barrier through the small open junctions and accumulate inside tumor tissues by the EPR effect [44,56].

Several recent studies have reported the design, optimization, and use of various types of liposomes as nanocarriers for PSs alone or combined with other chemotherapeutic agents, for effective cancer PDT [38,39].

#### 3.1.1. Tetraether Lipid-Based Liposomes

Tetraether lipids have longer lipophilic chains that contain ethers bonds, and hence, show high stability and lower susceptibility to oxidation. Thus, the use of tetraether lipid-based liposomes can increase liposomal membrane stability and integrity [45,56].

Curcumin, with an absorption spectrum of 300–500 nm, is a promising natural PS that could be used safely (at doses up to 12 g/kg/day) in PDT of superficial tumors. Reported studies demonstrate the ability of cancer cell lines to preferentially uptake a curcumin composite compared to normal cell lines. Upon its photoactivation, curcumin (in micromolar concentrations) produces capable of killing cancer cells to treat local superficial infections and cancers [57,58]. Duse et al. developed tetraether lipid-based (TELs) liposomes loaded with curcumin to kill cancer cells selectively [59]. Liposomes were designed by preparing a molar ratio of 90 DSPC:10 TELs using the thin-film hydration method followed by sonication at 56 °C. The curcumin-loaded liposomes had a size of about 208 nm, a zeta potential of −5.9 ± mV, % encapsulation efficiency (%EE) of 91%, and pronounced stability attributed to the tetraether lipids, which are reported to increase membrane stability and integrity. Furthermore, the curcumin liposomes showed the highest photocytotoxicity on SK-OV-3 ovarian carcinoma cells with a radiation fluence of 13.2 J/cm^2^ for the light-induced PDT (IC50 of 8.7 μM). Moreover, the prepared curcumin-loaded liposomes were found to have a minimal hemolytic effect (<40%) and a coagulation time of only 9.7 s compared to 112 s in the case of free curcumin [59].

The in vitro anticancer activities of the prepared curcumin-TEL liposomes were further investigated against cervical cancer cells and papillomavirus-related cancer cell lines [60]. The cancer cell lines were incubated with the designed liposomes at various concentrations ranging from 0 to 100 μmol/L for 4 h followed by LED irradiation at 457 nm for 45, 136, and 227 s at a fluence of 1, 3, and 5 J/cm^2^. The cytotoxic effects were then evaluated using the MTT, SYTO9/PI (propidium iodide), Annexin V-FITC (fluorescein isothiocyanate), clonogenic survival, and scratch (wound closure) assays against three different cancer cell lines (HeLa, UD-SCC-2, and VX2). The findings showed enhanced cytotoxicity at a light fluence of 3 J/cm^2^ (IC50 values of 9.52 μmol/L, 7.88 μmol/L, and 20.70 μmol/L against HeLa, UD-SCC-2, and VX2 cancer cell lines, respectively), reduced colony formation, proliferation, and cell migration rates. Liposomes loaded with curcumin was suggested as an efficient treatment of papillomavirus-associated cancers.

Plenagl et al. reported tetraether liposomes prepared with a molar ratio of 90 DPPC: 10 TELs encapsulating either hypericin (Hyp-TEL) using thin-film hydration method or hypericin-hydroxypropyl-β-cyclodextrin inclusion complex (HPCD-Hyp-TEL) through dehydration-rehydration vesicle technique [61]. The inclusion of hypericin in hydroxypropyl-β-cyclodextrin in liposomes prevents its photodegradation. The designed Hyp-TEL had an average particle size of 127 ± 14 nm, a zeta potential of −2 ± 1 mV, % encapsulation efficiency (%EE) of 82.5 ± 2.8. At the same time, the HPCD-Hyp-TEL had an average particle size of about 212 ± 13 nm, a zeta potential of −56 ± 1 mV, % encapsulation efficiency (%EE) of 81.6 ± 3.2. The %EE of hypericin was enhanced by increasing the liposomal membrane stability upon the addition of TEL [62]. The phototoxic effect of the prepared liposomes was evaluated on SK-OV-3 cancer cells using an LED fluence of 12.4 J/cm^2^. The IC50 values of Hyp-TEL and HPCD-Hyp-TEL were found to be 48 and 136 nM, respectively. Furthermore, considerable amounts of hypericin were uptaken by cancer cells as supported by confocal laser scanning microscopy results. The designed liposomes were hemocompatible and showed a coagulation time range of 5–12 s. These results support the use of integrated liposomal/PDT in cancer-targeted therapy.

Tetraether liposomes were also used to encapsulate protoporphyrin IX PS for vascular targeting and cancer treatment using PDT [63]. The liposomes, designed using 62 mol% TELs, had an average size of 170 nm and an average zeta potential −42 mV. A significant improvement of protoporphyrin IX phototoxicity (IC50 of 5 μM), when loaded in TEL liposomes and tested against SK-OV-3 cancer cells (irradiated with LED light of 672 mJ/cm^2^). On the other hand, the protoporphyrin IX-loaded TEL liposomes showed lower phototoxicity (IC50 of 12 μM) when tested on mouse fibroblasts.

Ali et al. reported developing different liposomal formulations accommodating temoporfin (second-generation, synthetic, effective PS) with enhanced phototoxicity against SK-OV-3 cancer cells [64]. Three liposomal formulations loaded with temoporfin (mTHPC) were prepared with molar ratios of 90 DPPC: 10 Cholesterol (DPPC/Chol/T), 95 DPPC: 5 DPPE–mPEG5000 (DPPC/DPPE mPEG5000/T), and 90 DPPC: 10 TEL (DPPC/TEL/T). The prepared liposomes had an average size range of 115 nm and zeta potentials ranging from −6.0 to −13.7 mV. The %EE was 78 ± 4, 81.7 ± 3, and 90 ± 3 % for DPPC/Chol/T, DPPC/DPPE mPEG5000/T and DPPC/TEL/T, respectively. The cell viability of SK-OV-3 cancer cells was reduced to 20% in the three liposomal formulations loaded with temoporfin when exposed to LED light of 10 J/cm^2^. Moreover, all the designed liposomal formulations exhibited hemocompatibility (<10% hemolysis) and a coagulation time <40 s.

#### 3.1.2. Stealth Liposomes

Stealth liposomes (PEGylated liposomes) are developed by the adsorption of PEGylated lipids (e.g., DPPE mPEG5000) on the liposomal surface, which prolongs the half-life of liposomes in circulation [65]. Steric hindrance occurs due to PEG reduces liposomal uptake by the reticuloendothelial system and their elimination via renal globular filtration. Moreover, the inclusion of the PEGylated lipids in the liposomal formulations improves their hydrophilicity, and hence, their shelf life [65].

In a different study, curcumin was loaded into liposomes comprising 9.5 HSPC: 0.5 DPPE mPEG5000 HCPC inhibits the leakage of curcumin outside the lipid membranes by imparting rigidity to the liposomal bilayer membrane [66]. The prepared liposomes exhibited significant phototoxic activity against skin cancer melanoma cells (MUG-Mel2) and squamous cell carcinoma (SCC-25) compared to free curcumin after exposure to LED light fluence of 2.5 J/cm^2^. The cytotoxicities were 53% (against MUG-Mel2) and 58% (against SCC-25) for curcumin-loaded liposomal mediated PDT compared to 27% (against MUG-Mel2) and 34% (against SCC-25) for free curcumin (10 µM) mediated PDT. The cytotoxic activity of the liposomal preparation against normal keratinocyte cells (HaCaT) was 11%, highlighting the safety of the designed liposomes. Flow cytometry showed that integration of PDT with liposome encapsulating curcumin increased apoptosis to 30% and 40% in MUG-Mel2 and SCC-25 cancer cells, respectively.

A study conducted by Corato et al. loaded iron oxide nanoparticles in the aqueous liposomal core and mTHPC PS in the lipid bilayer [67]. The dual-loaded liposomes were prepared with a molar ratio of 85 DPPC: 10 DSPC: 5 DPPE mPEG5000, which was then mixed with 3.3 mg/mL mTHPC and 0.7 M iron oxide NPs utilizing the reverse-phase evaporation approach. The prepared magneto-photoresponsive liposomes had a spherical structure, an average particle diameter of 150 nm, and one-month stability at 4 °C. The in vitro cell viability was assessed against SK-OV-3 cancer cells after exposure to either magnetic hyperthermia alone, PDT alone, or combined therapies. The % cell viabilities were 10% after exposure to magnetic hyperthermia alone, 5 and 1% after exposure to PDT alone (at 5 and 10 J, respectively), 0.2 and 0% after treatment with the liposomes loaded with iron oxide in the core and mTHPC in the aqueous layer/PDT (at 5 and 10 J, respectively). As supported by proteomic analysis, the dual-loaded liposomes seem to activate intrinsic apoptotic pathways by increasing ROS levels and mitochondrial damage.

Fisher et al. reported the design of lapatinib-loaded PEGylated liposomes for low-dose PDT of the invasive and resistant glioma [68]. Lapatinib is an antineoplastic, clinically approved PS that acts as an epidermal growth factor receptor (EGFR) inhibitor; however, it has poor penetration into glioma cells. PEGylated liposomes were prepared by mixing DPPC: DOTAP (1,2-dioleoyl-3-trimethylammonium-propane): cholesterol: DSPE-mPEG_2000_ at a molar ratio of 0.6:0.079:0.289:0.031. The prepared liposomes had an average particle diameter of 132 ± 9 nm, a zeta potential of 14.3 ± 0.8 mV, and %EE of 64.5 ± 8%. The moderately positive charge aids electrostatic attraction of liposomes to the anionic cells leading to increased lapatinib uptake. The phototoxicity of the nanoformulation loaded with lapatinib and ALA (the latter is metabolized in vivo to protoporphyrin IX) was assessed against human glioblastoma cancer cell lines (U87 and U87vIII) irradiated with 635 nm light provided by a laser at a light dose of 65 mW/cm^2^. A remarkable reduction (46%) in the LD50 in the case of lapatinib-loaded liposomes compared to the free lapatinib was observed.

Peng et al. designed long-circulating dual-loaded PEGylated liposomes loaded with Chlorin e6 PS and cisplatin, a first-generation platinum-based chemotherapeutic drug (PL-Ce6-Cis) [69]. In this context, the hydrophilic cisplatin was loaded in the aqueous liposomal core, while hydrophobic Ce6 was incorporated in the outer lipid bilayer. The dual-loaded PEGylated liposomes were prepared with a molar ratio of 10 DSPC:0.2 DSPE-PEG2000:5 cholesterol using the ethanol injection approach. A single dose of the dual-loaded liposomes integrated with light irradiation with a fluence of 100 J/cm^2^ could kill 80% of C26 colon cancer cells (compared to only 20% without irradiation), while maintaining minimal adverse toxic effects.

Another recent study reported the use of combined chemotherapy and PDT using dual-loaded PL-Ce6-Cis to treat malignant peripheral nerve sheath cancer (MPNST) [70]. In this regard, the cytotoxicity assessment was conducted against three different MPNST cancer cell lines (T265, ST8814, and S462-TY), derived from NF1 patients using a 662 nm diode laser with a power intensity of 95 mW. The cell viability was reduced significantly (<40%) for PL-Ce6-Cis compared to PL-Ce6 or PL-Cis. Moreover, the PL-Ce6-Cis dual-modal formulation was found to exert minimal neurotoxicity.

#### 3.1.3. Thermosensitive Liposomes (TSLs)

Thermosensitive liposomes (TSLs) are used for triggered release delivery into solid tumors, with at least one formulation currently under phase III clinical trials [71,72,73,74]. Exposure to mild hyperthermia (39–43 °C), causes a phase change of the liposomal lipid bilayer from a solid gel well-arranged phase (L_β_) to a fluid tangled phase (L_α_). This results in enhanced membrane permeability, thus facilitating the release of the liposomal cargo into cancer cells [75,76,77]. It is of note that the lipid composition of liposomes is key in the TSLs temperature-triggered drug release [78]. Drug release occurs at temperatures equal or lower (by 1–2 °C) to the melting phase transition temperature (Tm) of lipids where structural deformations in the lipid membrane increase the membrane permeability of the lipid bilayer, facilitating the release of the liposomal drug content [79,80]. DPPC, for instance, has a Tm slightly higher than normal body temperature (about 41 °C), and is, thus, used for TSL systems [78].

Shemesh et al. developed an integrated thermosensitive liposomes (TSLs)/PDT encapsulating indocyanine green PS (ICG) to treat triple-negative breast cancer [81]. The TSLs are designed by mixing a molar ratio of 100 DPPC:50 SoyPC (l-α-phosphatidylcholine):30 Chol:0.5 DSPE-PEG 2000 using the thin-film hydration method. The prepared TSLs had an average particle diameter of 71 ± 10 nm and could encapsulate ~500 μM of ICG. The TSLs loaded with ICG (37.5 μM) demonstrated significant cytotoxicity (cell viability < 20%) against MDA-MB-468 and HCC-1806 cells at laser radiation of 14 J/cm^2^ compared to free ICG [81].

**Figure 4:**
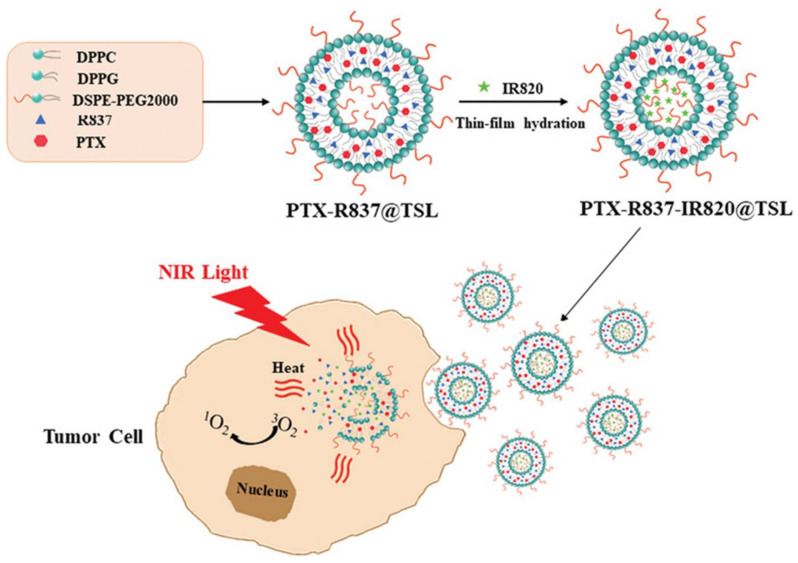
Thermosensitive liposomes loaded with IR820 PS, paclitaxel, and imiquimod (R837) [81]. DPPC, 1,2-Dipalmitoyl-sn-glycero-3-phosphocholine; DPPG, 1,2-Dipalmitoyl-sn-glycero-3-phosphoglycerol, sodium salt; 1,2-distearoyl-sn-glycero-3-phosphoethanolamine-N-[amino(polyethylene glycol)-2000] (ammonium salt); R827, imiquimod; PTX, Paclitaxel; IR820, photosensitizer. Reprinted with permission from ref. [82]. 2019 Meng et al.

**Figure 5:**
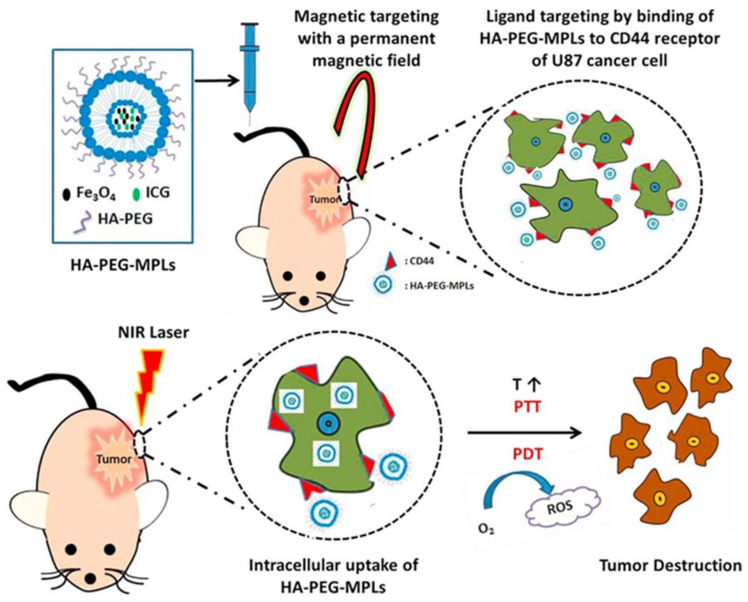
Schematic illustration of HA-PEG coated magnetic/photoresponsive liposomes for combined photothermal/photodynamic cancer therapy. ICG, Indocyanin green PS; HA-PEG, hyaluronic acid-polyethylene glycol; MPLs, magnetic/photoresponsive liposomes; PTT, photothermal therapy; ROS, reactive oxygen species. Reprinted with permission from ref. [87]. 2019 Meng et al.

Meng et al. combined chemotherapy, immunotherapy, and PDT to treat gastric cancer [82]. Thermosensitive liposomes were loaded with IR820 PS, paclitaxel anticancer drug, and imiquimod (R837) (Figure 4). IR820 PS is derived from ICG and is more stable, less expensive, and suitable for photothermal and photodynamic therapies [83,84]. Imiquimod (R837), a dendritic cell activator, is an agonist to TLR7 and has immunomodulatory activities [85]. Mouse fore-stomach carcinoma cell (MFC) treated with the designed liposomes and irradiated with 808 nm light provided by a laser at a light dose of 2.5 W/cm^2^ showed that the cell viability decreased to 10% (Figure 5).

#### 3.1.4. Miscellaneous Liposomes

A study reported developing a novel multimodal delivery system, lipopolyplexes (LPPs) loaded with curcumin, combined with PDT for improving gene delivery to SK-OV-3 cancer cells [86]. LPPs used comprised cationic polyethyleneimine and luciferase-expressing pCMV-luc plasmid with anionic liposomes in a ratio of 1:0.5. Liposomes were formulated with a molar ratio of 70 DOPE:15 DPPC:15 cholesterol. The designed LPPs loaded with curcumin were spherical and had a particle size of 200 nm and zeta potential of +8.6 mV ± 1.7 mV. Treatment of SK-OV-3 cells with LPPs loaded with curcumin and irradiation with LED light at 457 nm (irradiation fluence of 1 J/cm^2^) resulted in a significant increase in luciferase expression. The designed LPPs had minimal hemolytic effects and plasma coagulation time of 32 s.

Another recent study reported PDT combined with curcumin-loaded magnetic/photoresponsive liposomes (MPLs) in treating papillomavirus-related cancers (Figure 5) [87]. Citric acid-coated iron oxide (CMNPs) magnetic nanoparticles are synthesized employing the chemical precipitation method [88]. Liposomes were prepared using thin-film hydration technique and lipid mixtures in a molar ratio of 5 DSPC: 3 Chol: 1 DDAB (1,2-stearoyl-sn-glycero-3-phosphocholine, dimethyl octadecyl ammonium bromide). In the hydration step, the thin film was hydrated with 0.3 mg/mL CMNPs, 50 µg/mL ICG, producing the ICG-loaded magnetic liposomes. Liposomes were then coated with hyaluronic acid-polyethylene glycol (HA-PEG) PS via sonication. Interactions between anionic HA and the cationic DDAB were responsible for the self-assembly of HA-PEG on the surface of MPLs. HA binds to the over-expressed CD44 receptors on the cell surface of human primary glioblastoma cancer cells (U87MG). The designed HA-PEG MPLs had an average particle size of about 220 nm and exhibited significant cytotoxicity (cell viability of ~30% at a concentration of 2 mg/mL) against U87MG after exposure to near-infrared laser radiation of 2 W/cm^2^. Moreover, a xenograft tumor model designed by subcutaneous implantation of U87MG cells in nude mice showed the accumulation of HA-PEG MPLs in cancerous tissues. Following laser irradiation, the tumor size was reduced by 13% compared to the control (Figure 5).

Another recent study reported the encapsulation of ICG in chitosan-coated liposomes for PDT of melanoma. Chitosan coating has imparted stability to liposomes and improved their cellular uptake into B16-F10 melanoma cancer cells [89]. The liposomes were designed with a molar ratio of 16 DMPC: 3 Chol using the modified freeze-drying method. The positively charged chitosan (0.1%) coated the anionic liposomal surface through electrostatic interactions. The prepared chitosan-coated liposomes loaded with ICG had a mean droplet size of 1983 ± 270 nm, a zeta potential of 43.2 ± 1.2, and a loaded ICG concentration of 1.19 ± 0.03 mg/mL. Cellular uptake of ICG was dramatically increased (owing to the cationic nature of chitosan coats). Moreover, the prepared liposomes showed remarkable phototoxicity against B16-F10 cancer cells after exposure to laser (775 nm) at a power of 0.23 mW for 2.5 min.

A study conducted by Wu et al. reported the preparation of zwitterionic liposomes encapsulating methylene blue (MB) PS that can generate ROS after light exposure and causing cancer cell apoptosis [90]. MB has poor penetration into cancer cells; hence, this study aimed to encapsulate MB into zwitterionic liposomes to protect loaded drugs from degradation, prolong systemic circulation, and enhance cellular uptake. A zwitterionic polymer-lipid poly(12-(methacryloyloxy) dodecyl phosphorylcholine) was self-assembled with DSPC in a molar ratio of 1:4. The prepared zwitterionic liposomes had an average particle size of 150 nm and fast release rates (about 90% of the MB was released over 8 h). A significant increase in the ROS release was observed in the case of MB-loaded liposomes compared to the unloaded MB. Increased cytotoxicity of the MB-loaded zwitterionic liposomes was observed against breast cancer cells (4T1 cells) compared to unloaded MB irradiated with LED light (660 nm) of 165 mW for 6 min. Annexin V-FITC assay showed that cytotoxicity took place via apoptosis.

Finally, Rizvi et al. prepared two liposomal formulations encapsulating Visudyne or a lipid conjugate of BPD. These formulations were combined to localize the PSs and cause photodamage (following irradiation with a 690 nm diode laser at a light dose of 50 mW/cm^2^) to mitochondria, endoplasmic reticulum, and lysosomes in a 3D model of ovarian cancer [91]. The recent studies in integrating liposomes with PDT in cancer treatment are summarized in Table 3.

## 4. Conclusions

In this paper, we reviewed the most recent state-of-the-art studies concerning the use of integrated liposomes/PDT for effective cancer PDT. The ability of liposomes to accommodate hydrophilic and hydrophobic photosensitizers in the aqueous interior and the outer lipid bilayer, respectively, make them top candidates for the delivery of PSs in cancer PDT. Besides, liposomes can target the loaded PSs to cancer cells via active or passive targeting. Moreover, liposome surfaces could be decorated with particular ligands that recognize and bind to overexpressed receptors and proteins in tumor tissues, such as folate, estrogen, spermine, and galactose receptors. Liposomes were also found to improve the selective targeting of PS alone or combined with other chemotherapeutic drugs, hence minimizing their toxic effects. Several studies have reported the enhanced effectiveness of PDT upon encapsulation of PSs in different liposomal formulations. Therefore, these combinatorial/multimodal platforms are likely promising candidates that can improve the effectiveness of cancer PDT.

## Figures and Tables

**Table 1:** Chemical structure and charge of the most common lipids used in liposomal formulations.

Lipid	Name	Chemical Structure	Charge
DSPC	1,2-Distearoyl-*sn*-glycero-3- phosphocholine	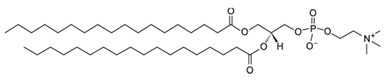	Zwitterion
DSPE	1,2-Distearoyl-*sn*-glycero-3-phosphorylethanolamine	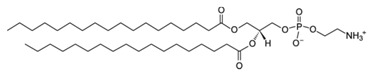	Zwitterion
DPPC	1,2-Dipalmitoyl-*sn*-glycero-3 -phosphocholine	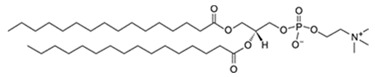	Zwitterion
HSPC	L-α-Phosphatidylcholine, hydrogenated (Soy)	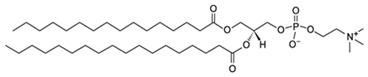	Zwitterion
DOPC	1,2-Dioleoyl-*sn*-glycero-3- phosphocholine	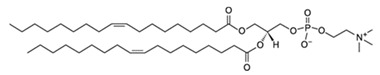	Zwitterion
EPC	1,2-Dipalmitoyl-*sn*-glycero-3-ethylphosphocholine (chloride salt)	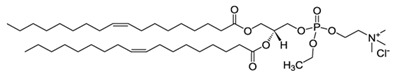	Zwitterion
DSPG	1,2-Distearoyl-sn-glycero-3- phosphoglycerol, sodium salt	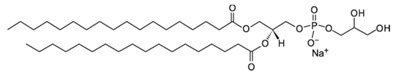	Anionic
Chol	Cholesterol	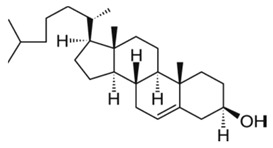	Neutral
DPPE mPEG5000	1,2-Distearoyl-sn-glycero-3-phosphoethanolamine-N-[methoxy (polyethylene glycol)-2000] (sodium salt)	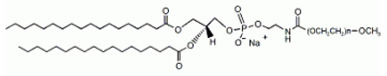	Anionic

**Table 2:** FDA-approved liposomal-based formulations in cancer treatment [52].

Name	Approval Year	Lipid Composition	Chemotherapeutic Drug	Clinical Use
Doxil^®^	1995	HSPC, Cholesterol, and PEG 2000-DSPE	Doxorubicin	Ovarian and Breast cancers
DaunoXome^®^	1996	DSPC and Cholesterol	Daunorubicin	Kaposi’s Sarcoma
Depocyt^®^	1999	DOPC, DPPG, Cholesterol and Triolein	Cytarabine/Ara-C	Neoplastic meningitis
Myocet^®^	2000	EPC and Cholesterol	Doxorubicin	Metastatic breast cancer in combination with cyclophosphamide
Mepact^®^	2004	DOPS and POPC	Mifamurtide	Non-metastatic osteosarcoma
Marqibo^®^	2012	SM and Cholesterol	Vincristine	Acute leukemia
Onivyde™	2015	DSPC: DPPE mPEG5000	Irinotecan	Metastatic pancreatic cancer

**Table 3:** Encapsulation of PSs into liposomes in cancer PDT.

Liposomes		Liposomal Formulation	PS	Light Dose	Tumor Type	Outcomes	Ref
Tetraether liposomes		DSPC TELs	Curcumin	13.2 J/cm^2^	Ovarian cancer (SK-OV-3)	- Enhanced photocytotoxicity. - Minimal hemolytic effect.	[59]
		DSPC TELs	Curcumin	1, 3, and 5 J/cm^2^	Papillomavirus-related cancer cell lines	Enhanced cytotoxicity, reduced colony formation, proliferation, and cell migration rates.	[60]
		DPPC TELs	Hypericin	12.4 J/cm^2^	SK-OV-3	- Enhanced photocytotoxicity. - High cancer cell uptake. - Hemocompatibility.	[61]
		TELs	Protoporphyrin IX	672 mJ/cm^2^	SK-OV-3	- Improvement of phototoxicity on cancer cells while maintaining lower phototoxicity on normal cells.	[63]
		DPPC TELs	Temoporfin (mTHPC)	10 J/cm^2^	SK-OV-3	- Enhanced cytotoxicity. - Hemocompatibility.	[64]
Stealth Liposomes		DPPC DPPE-mPEG5000	Temoporfin (mTHPC)	10 J/cm^2^	SK-OV-3	- Enhanced cytotoxicity. - Hemocompatibility.	[64]
		HSPC DPPE mPEG5000	Curcumin	2.5 J/cm^2^	Skin cancer melanoma cells (MUG-Mel2) and squamous cell carcinoma (SCC-25)	- Enhanced cytotoxicity on cancer cells. - Lower cytotoxicity on normal keratinocyte cells.	[66]
		DPPC DSPC: DPPE mPEG5000	mTHPC	5 and 10 J/cm^2^	SK-OV-3	Enhanced cytotoxicity on cancer cells.	[67]
		DPPC DOTAP	Lapatinib	65 (mW/cm^2^)	Human glioblastoma cancer cell lines (U87, U87vIII)	Remarkable reduction in the LD50.	[68]
		DSPC DSPE-PEG2000 Cholesterol	Chlorin e6	100 J/cm^2^	C26 colon cancer cells	- Kills 80% of the C26 colon cancer cells. - Minimum adverse reactions.	[69]
		DSPC DPPE mPEG5000Cholesterol	Chlorin e6	95 mW/cm^2^	Malignant peripheral nerve sheath cancer	- Enhanced cytotoxicity on cancer cells. - Minimal neurotoxicity.	[70]
Thermosensitive liposomes		DPPC SoyPC Chol DSPE-PEG 2000	ICG	14 J/cm^2^	Triple-negative breast cancer (MDA-MB-468 and HCC-1806 cells)	Significant cytotoxicity compared to free PS	[81]
		DPPC DPPG DPPE mPEG5000	IR820	2.5 W/cm^2^	mouse fore-stomach carcinoma cell (MFC)	Significant cytotoxicity	[82]
Miscellaneous Liposomes	Lipopolyplexes (LPPs)	DOPC DPPC Cholesterol	Curcumin	1 J/cm^2^	SK-OV-3	- Significant enhancement in the luciferase expression of SK-OV-3. - Biocompatibility.	[86]
	Magnetic/photo-responsive liposomes	DSPC Cholesterol DDAB	Indocyanine green (ICG)	2 J/cm^2^	Glioblastoma cancer cells (U87MG)	Significant cytotoxicity and accumulation in cancer cells.	[87]
	Chitosan-coated liposomes	DMPC Cholesterol	ICG	250 mW	Melanoma	Improved permeability and phototoxicity.	[89]
	Zwitterionic liposomes	Poly-(12-meth-acryloyloxy)-dodecyl phosphoryl-choline DSPC	Methylene blue (MB)	165 mW	Breast cancer cells (4T1 cells)	- Enhanced cytotoxicity on cancer cells. - Improved safety.	[90]
